# Internationalizing Medical Education: The Special Track Curriculum 'Global Health' at Justus Liebig University Giessen

**DOI:** 10.3205/zma000994

**Published:** 2015-11-16

**Authors:** Michael Knipper, Adrian Baumann, Christine Hofstetter, Rolf Korte, Michael Krawinkel

**Affiliations:** 1 Justus Liebig University Giessen, Institute for History of Medicine, Giessen, Germany; 2 Justus Liebig University Giessen, Medical Students, Giessen, Germany; 3 Justus Liebig University Giessen, Institute of Hygiene and Environmental Medicine, Giessen, Germany; 4 Justus Liebig University Giessen, Institute of Nutritional Science, Giessen, Germany

**Keywords:** internationalization, undergraduate medical education, global health, mobility, cultural competence

## Abstract

Internationalizing higher education is considered to be a major goal for universities in Germany and many medical students aspire to include international experiences into their academic training. However, the exact meaning of “internationalizing” medical education is still poorly defined, just as is the possible pedagogic impact and effects.

Against this background, this article presents the special track curriculum on global health (in German: *Schwerpunktcurriculum Global Health*, short: *SPC*) at Justus Liebig University Giessen, which was established in 2011 as a comprehensive teaching program to integrate international perspectives and activities systematically into the clinical years of the medical curriculum. The report of the structure, content, didactic principles and participants’ evaluations of the SPC is embedded into a larger discussion of the pedagogic value of a broad and interdisciplinary perspective on “global health” in medical education, that explicitly includes attention for health inequities, social determinants of health and the cultural dimensions of medicine and health abroad and “at home” (e.g. in relation to migration). We conclude that if properly defined, the emerging field of “global health” represents a didactically meaningful approach for adding value to medical education through internationalizing the curriculum, especially in regard to themes that despite of their uncontested value are often rather weak within medical education. The concrete curricular structures, however, have always to be developed locally. The “SPC” at Giessen University Medical School is only one possible way of addressing these globally relevant issues in one particular local academic setting.

## Zusammenfassung

Internationalisierung gilt als Zukunftsaufgabe für die deutschen Hochschulen, und Auslandsaufenthalte sind unter Medizinstudierenden weit verbreitet. Eine systematische Verknüpfung internationaler Elemente in der medizinischen Ausbildung findet bisher jedoch kaum statt. Auch ist bislang nicht geklärt, was der Begriff „Internationalisierung“ im Medizinstudium konkret bedeutet und bewirken kann. Dieser Projektbericht stellt dazu das 2011 an der JLU Gießen etablierte „Schwerpunktcurriculum Global Health“ (SPC) vor: ein Lehrprojekt zur systematischen Integration internationaler Elemente im klinischen Abschnitt des Medizinstudiums. Das umfassende Themenfeld „Global Health“ wird aufgrund der explizit „globalen“ Perspektive, die auch die Medizin in Deutschland einschließt, als didaktisch sinnvoller Zugang für eine „Internationalisierung“ des Medizinstudiums zur Diskussion gestellt. Das Potential für die medizinische Ausbildung zeigt sich vor allem für disziplinübergreifende Themen und Kompetenzen, die im Regelstudium oft nur punktuell behandelt werden. Beispiele sind: soziale Determinanten von Gesundheit, Gesundheitssysteme und -politik, Medizinethik, Menschenrechte, kulturelle Kompetenz, sowie Reflexionsfähigkeit und ein differenziertes Verständnis der kulturellen, sozialen und historischen Bedingtheit von Medizin und ärztlichem Handeln in Deutschland und global.

## Introduction

Internationalizing higher education is officially considered to be a major goal for German universities ([16]:11). This message from the German Rectors' Conference (*Hochschulrektorenkonferenz*, HRK) in 2008 has long been internalized by medical students. According to statistics of the German Academic Exchange Service (*Deutscher Akademischer Austauschdiennst*, DAAD), about one third (29%) of medical students took part in the ERASMUS program, in clinical traineeships, research trips, electives or even spent a whole semester or more abroad in 2013 [13]. At the same time, students demand more learning opportunities in the emerging field of „global health“ ([6], [7], [9], [29]). Yet the actual integration of international activities and perspectives within the medical curriculum is still rather weak [29]. International clinical clerkships, for example, are usually organized on an individual basis and without systematic integration into the student’s training. Systematic preparation for going abroad and pedagogically oriented wrap-up afterwards are rare. Students themselves have to care for getting prepared, either by attending seminars held by the German Association of Medical Students (*Bundesvertretung der Medizinstudierenden in Deutschland*, bvmd [http://www.bvmd.de]), or events organized by insurance companies or financial service providers who combine this kind of training with promotional activities among the future physicians. What kind of professional, social or cultural competencies are being obtained during a stay abroad thus remains a matter of chance. 

Internationalizing medical education should, however, mean more than gaining clinical experiences under exotic conditions, sometimes even including the exploitation of poverty and precarious health systems in countries of the global south for training practical skills. How can the benefits for medical education actually look like, that are supposed to be achieved through “international exchange of science and culture“([16]:16) as suggested by the HRK? How can internationalization be operationalized for medical studies? The aim of the present article is to discuss these questions at the example of the special track curriculum on Global Health for undergraduate medical students, established at the Faculty of Medicine of the University of Giessen in Germany in 2011 (*Schwerpunktcurriculum Global Health*, SPC). The SPC is a comprehensive teaching project for medical students in the four clinical years of their academic training (3^rd^ to 6^th^ year), with particular interest in Global Health. It links internationally oriented elements of the compulsory curriculum with electives and academic experiences abroad as well as newly developed courses specific to the SPC [http://www.uni-giessen.de/cms/fbz/fb11/studium/medizin/klinik/spc/spc-global]. Our discussion about the possible content, meaning and educational impact of internationalizing medical education is guided by concept of Stütz and colleagues [29], which highlights three core elements of internationalization: international mobility, global health and cultural competences. 

## Project Description

### Global Health 

The term *Global Health* describes a broad and heterogeneous subject area that focuses on the multiple challenges for medicine and health care in an increasingly interconnected world. There is no universal definition of Global Health available yet, and will probably never be, due to the variety of backgrounds, perspectives and interests of the different actors and institutions engaged in this emerging field. Supported by selected international publications (cf. [1], [4], [6], [5], [15], [24]), the working definition of “Global Health” applied in the SPC at the University of Giessen basically consists of three core elements:

- As suggested already by the very notion, “Global Health” implies the commitment to improve health and access to health care globally, and the reduction of health related inequities, according to the “human right to health” ([30], also: [10]).
- The second feature is the geographically ‘global’ orientation: (cf. [5]): In line with the strategy for internationalizing higher education issued by the HRK [16], the global-health-perspective includes attention for health inequities and health related consequences of processes related to globalization not only in the south but also in the northern hemisphere, including migration and climate change. Priority issues of the international health agenda like „social determinants of health“ [25], „non-communicable-diseases“ [http://www.who.int/mediacentre/factsheets/fs355/en/], as well as the human right to health, are considered both in the global north and south, in African countries as well as in Europe, North America or Central Asia. Global Health explicitly transcends the geographically confined focus of “international health“, “tropical medicine“, and an exclusive view on “development cooperation”.
- Third, interdisciplinary cooperation is of upmost importance for Global Health: Social sciences, anthropology, history, ethics, and the medical humanities as a whole, as well as economics, political sciences and law are essential and have to be put into close and meaningful relation with clinical disciplines, epidemiology and public health. Clinical, quantitative and natural science based approaches alone are unable to address the social, historical, legal and economic backgrounds as well as social and cultural dimensions of health, medicine and medical care, and to understand the complex dynamics between global factors and the local realities of health, suffering and care ([1], [4], [15]).

#### Global Health in Medical Education 

On national and international level, Global Health is an issue of growing importance in medical education (cf. [6], [7], [8], [9], [14], [18], [29]). One particular argument for the implementation of the SPC Global Health at the University of Giessen was the huge didactic potential of this evolving field for medical education. In our opinion, Global Health is not a completely new and separated subject with particular content and learning objectives alongside other specialist disciplines that needs to be added to existing medical curricula. And the overall intention for teaching Global Health is not to prepare students for long-term careers abroad, e.g. in development cooperation or international organizations. In contrast, teaching Global Health deepens and reinforces the content and learning objectives of a variety of clinical and theoretical subjects through international and cross-cultural comparison and interdisciplinary cooperation (cf. [16], [29]). Students are prompted and encouraged to address medical questions transcending national, cultural and disciplinary boundaries and modes of thought. Important topics of the general medical curriculum are put into a wider context and addressed from a perspective that gives priority to equity and the social, cultural, political and other dimensions of health and disease beyond and behind the clinical and technical aspects. Examples are disease control and prevention (e.g. tobacco, obesity), nutrition and food security, and cultural competence, communication (physician-patient as well as within professional teams), medical ethics and human rights. International comparison as well as the interaction with patients perceived to be culturally different in Germany, can be used didactically for directing the students’ attention to the socio-economic, cultural and structural preconditions of medicine and health care that usually tend to be seen as self-evident or taken for granted. Global Health can be used as a “magnifying glass” ([23]: 3) in medical education, to improve the “visual acuity” of students and teachers related to these important dimensions of medicine. According to a statement of the German Association of Medical Students in 2009, Global Health in medical education is simply to “keep pace” [6] with the accelerated dynamics that shape medicine and health today. 

#### Structure of the Special Track Curriculum (SPC) Global Health 

In general terms, “special track curricula” (*Schwerpunktcurricula,* SPC) are educational structures developed at the Medical Faculties of the neighboring Universities Giessen and Marburg that aim to provide the students in the clinical years of the undergraduate medical curriculum (3^rd^ to 6^th^ year) with a possibility to complement their training with additional content, according to individual thematic priorities. Besides Global Health, the University of Giessen offers special track curricula in the fields of “Pediatrics” (in cooperation with Marburg University), “Musculoskeletal System“ as well as “Anesthesiology and Intensive Care“. 

The SPC Global Health is basically an additional curriculum with 2 to 3 hours of classroom teaching per week over an average time span of five semesters. It consists of 9 modules, including elective courses that existed already previously (“Tropical Medicine and International Health”, “Migration and Health”), additional seminars of changing content to dig deeper in specific topics (like “Health Systems and Financing”, “Maternal Health”, or “Non-Governmental Organizations and Global Health”), and lecture series (see table 1 (Tab. 1)). Students are invited to include lectures, summer schools and further academic activities offered by other universities in Germany and abroad. At least one academic stay abroad (clinical clerkship, exchange semester, etc.) is also required, and the participants have to present an oral report of this experience in one of the SPC-courses, with a thematic focus on one particular issue of the broad field of Global Health. Moreover, the participants have to write and submit a certain amount of reports about presentations and lectures offered within the SPC Global Health or by another academic institution at home or abroad. For giving all students the possibility to present, and for encouraging and continuously training the creative application of the Global Health perspective transmitted in the classroom, every semester one “international afternoon” is organized: German students present their experiences abroad, while international students visiting Giessen talk about their experiences living in Germany and getting involved with Germans.

Due to reasons of capacity, a maximum of 15 students can enter the program each semester, after a careful selection process. A number of 123 students is currently enrolled in the program (April 2015), with 15 graduates as yet. 

A “student advisory board” war established since the beginning, in order to encourage students to engage in the development of content, structure and management of the SPC, as well as in the process of continuous evaluation: 2 to 5 volunteers function as a connection between students and faculty, to collect suggestions, critical remarks and other forms of formal and informal feedback of their fellow students that is needed for the further development and quality assurance of the program. The members of the student advisory board also organize and chair events like the “international afternoon“, supported by faculty members.

#### The Didactic Concept of the SPC Global Health 

**Figure 1:**
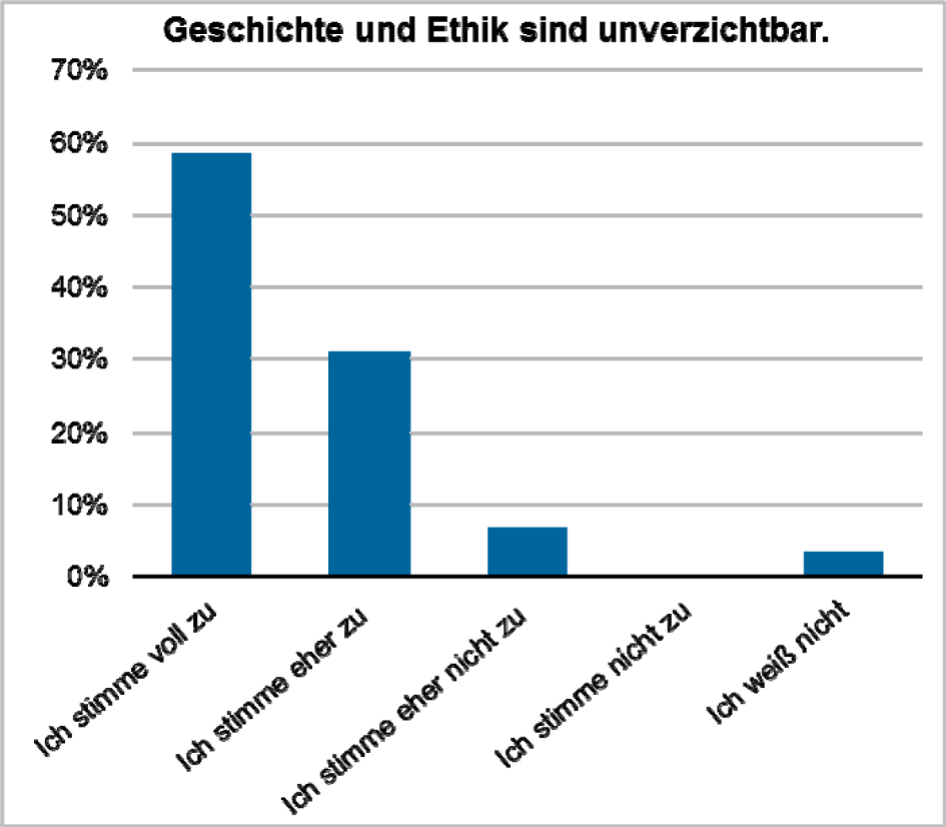
Approval of the expression that „history and ethics are indispensable in teaching health economics and systems” (“fully agree” on the left to “disagree” and “no opinion” on the right, absolute numbers: 17/9/2/0/1). Results of a compulsory final assessment and evaluation (online, anonymous) on a special seminar on “health systems and financing”, summer term 2012 (n=29, response rate 100%).

The didactic concept can be characterized by the following points:

1. The SPC is designed as a **reflective portfolio**: Throughout the curriculum, the students collect certificates, reports and written assignments. At two points in time – after completing two or three active semesters and at the end of the curriculum – they participate in feedback discussions with faculty and fellow students where they present what they have already done and learned, what they missed and what their doubts, thoughts and conclusions are regarding Global Health in general, and compared to their expectations at the moment when they entered the SPC. The aim of these meetings is to foster reflection and coherence in the learning process, and to contribute to the ongoing evaluations and further development of the program.
2. **Interdisciplinary approach: **According to the particular topic of a course or lecture, clinical, biomedical and the perspectives of other disciplines such as cultural and social anthropology, law and political sciences are addressed as explicit as possible. Faculty and invited speakers are encouraged not only to reflect, in their presentations, the current “state of the art” of the disciplines they present, but also to give particular attention to and explain the theoretical approaches and methodologies deployed. Students highly appreciate when guest speakers from Germany and abroad convey concrete insights in their daily work (e.g. at the WHO) and/or research projects.
3. **Medical Humanities:** The Giessen SPC is characterized by a strong involvement of the medical humanities, like history, anthropology and ethics, literary and linguistic studies but also art and literature. We encourage and guide students to develop creative ways of expressing their observations, questions and thoughts (e.g. internet blogs, videos, creative writing [3], [26], [27]). In regard to intercultural relations and „cultural competence“, medical anthropology and especially the principles of ethnography are conveyed as essential perspectives and tools for a thought- and meaningful approach to culture, ethnicity, and cultural diversity in the field of medicine (cf. [19], [20], [21], [27]). In course evaluations and feedback discussions, students often express high esteem for the perspectives of medical anthropology and history for global health (see figure 1 (Fig. 1) and [22]).
4. **The didactic use of international academic experiences: **International academic activities, like clinical clerkships, internships or exchange semesters, are a central didactic element of the SPC Global Health. Going abroad offers students the chance to experience, observe and analyze theoretical aspects and abstract course contents (like social determinants of health, health inequities, health systems and financing) in a setting different than at home. For systematic observation and reflection, a small guideline was developed [http://www.uni-giessen.de/cms/fbz/fb11/studium/medizin/klinik/spc/spc-global/downloads/Bleitf3] and preparative seminars are offered to students for discussing ethical, social and cultural challenges when going abroad. The international experience is thus embedded in a pre-departure seminar and an oral report in one of the courses of the SPC, with observations and reflections inspired and guided by reports and feedback of fellow students and faculty, and by the guideline.
5. **Blended learning: **The combination of classroom teaching, digital media and virtual courses is well established in the SPC. All forms of media are used and combined thoughtfully, from classic text books, monographs and articles, lectures and discussion rounds, to digital media, videos and e-learning tools. In spring 2014, a very successful seminar war organized in cooperation with HarvardX, combining the SW25x *Massive Open Online Course* (MOOC) on Global Health with compulsory classroom sessions and a final assessment and evaluation [22]. On the initiative of students, this first experiment was followed by another online course blended with class room sessions on *“Medical Peace Work*” [http://www.medicalpeacework.org/].

#### Evaluation and Quality Assurance 

The SPC as a whole as well as all teaching units undergo constant evaluation and further development. Basic elements of the evaluation strategy are online questionnaires, the feedback provided by the members of the student advisory board, and especially the interim and final feedback sessions with all students according to the “reflective portfolio” approach presented above. The questionnaires for the anonymous online evaluation usually contain typical quantitative questions (e.g. final grade, estimation of the balance between investment and results, fulfillment of expectations etc.) and open questions on content and for collecting individual responses. The aim is to get meaningful data on all dimensions of the courses and the whole program (content, achievement and development of learning objectives, performance, relevance, etc.). Moreover, in seminars that focus on new topics or apply especially innovative perspectives (e.g. from the medical humanities), participants are being asked for their opinions and suggestions regarding the integration of this content or teaching methods into the compulsory curriculum. 

**Figure 2:**
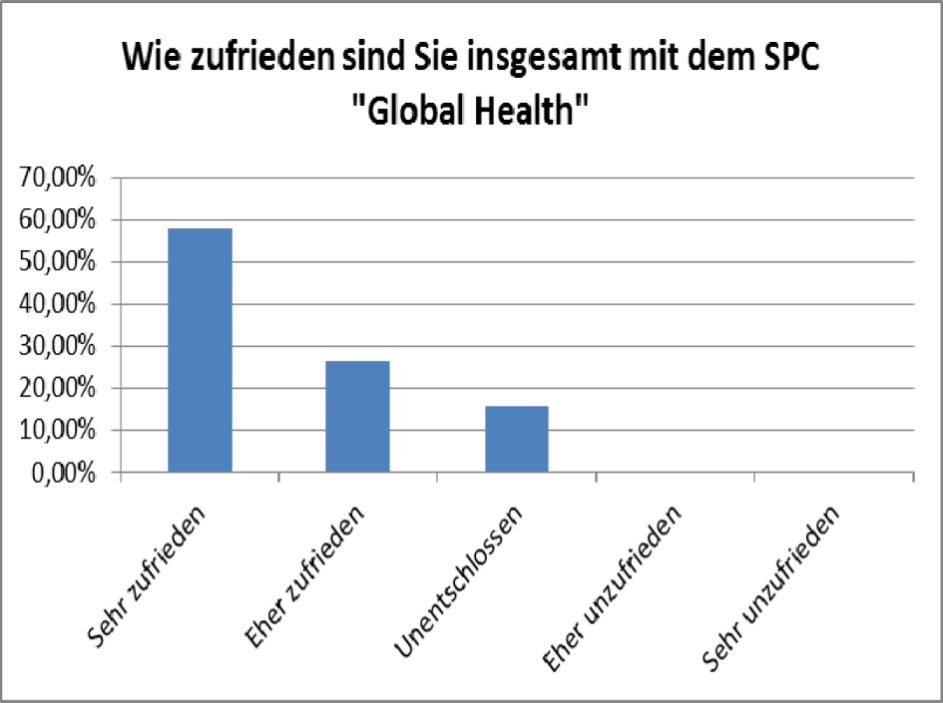
General satisfaction (from „highly satisfied“ left to “not satisfied” on the right). Results of the general evaluation of the SPC Global Health, winter term 2011/12 (voluntary online-survey with at that time, 33 active participants, n=19, response rate 59%)

**Figure 3:**
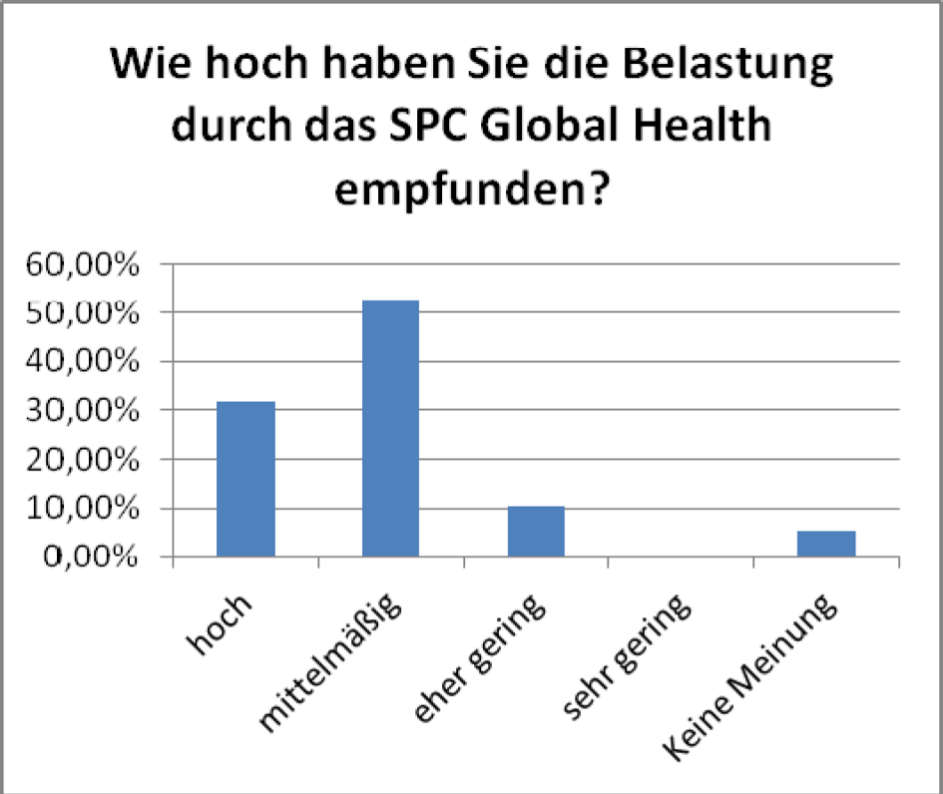
Perception of work load (from „huge“ on the left to „little“ and “no opinion” on the right). Results of the general evaluation of the SPC Global Health, winter term 2011/12 (voluntary online-survey with at that time, 33 active participants, n=19, response rate 59%)

**Figure 4:**
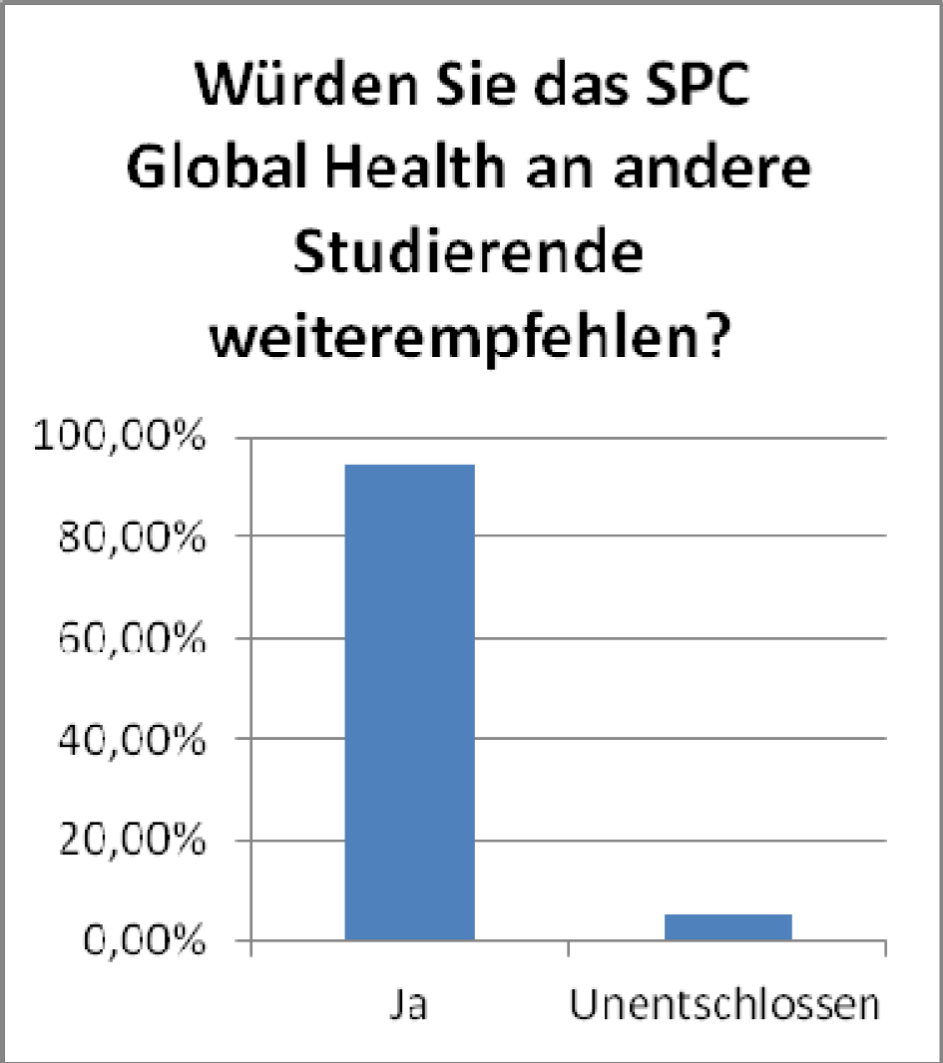
Likelihood to recommend the SPC to fellow students (18 „yes“, 1 „no opinion”, no negative response). Results of the general evaluation of the SPC Global Health, winter term 2011/12 (voluntary online-survey with at that time, 33 active participants, n=19, response rate 59%)

**Figure 5:**
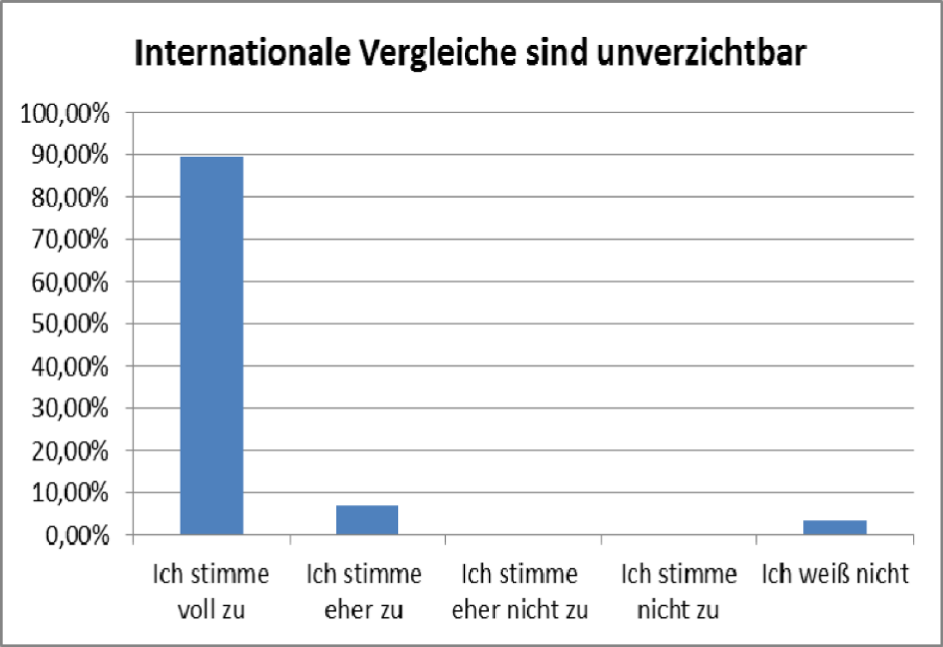
Approval of the expression that an international comparative approach is “indispensable” (“fully agree” on the left to “disagree” and “no opinion” on the right, absolute numbers: 26/2/0/0/1). Results of a compulsory final assessment and evaluation (online, anonymous) on a special seminar on “health systems and financing”, summer term 2012 (n=29, response rate 100%).

Exemplary evaluation results are presented in figure 1 (Fig. 1), figure 2 (Fig. 2), figure 3 (Fig. 3), figure 4 (Fig. 4), and figure 5 (Fig. 5); characteristic quotes from answers to open questions in table 2 (Tab. 2). 

## Discussion

In line with the internationalization strategy of the HRK and Giessen University itself, the SPC Global Health pursues an educational strategy to integrate „international elements and intercultural dimensions“ ([16]: 32) into medical education. The overall goal is to interconnect international activities systematically. With regard to the three core elements suggested by Stütz and colleagues [29] – international mobility, global health and cultural competences – at least those students who actively participate and pursue the complete SPC receive this kind of a coherent and comprehensive educational offer. International mobility is important yet not an exclusive means for “internationalizing” medical education, just as the global health perspective and cultural competence are also relevant within the home country (in this case Germany): Topics like migration and diversity both illustrate the need and offer multiple possibilities for applying an “international lens” and deploy a properly defined global health perspective in medical education “at home”. 

But does the SPC Global Health actually comply with the comprehensive concept of “internationalizing medical education” presented above? This question will be discussed in relation to the following three points:

1. How can the didactic value („additional benefit“) of internationalizing medical education be specified?
2. To what extent has the systematic approach towards internationalization been integrated into the general curriculum at Giessen University Medical School?
3. How can the model presented in this article be transferred to other universities?
4. According to the first experiences with the SPC Global Health, the additional benefit of internationalizing medical education consists mainly in the following aspects: It draws attention to the social, cultural, historical, political, economic and legal aspects of medicine and health, and thus trains transdisciplinary thinking, communication and collaboration. Many students explicitly demand and appreciate a broader view on health and medical care that complements the rather narrow focus of clinical and biomedical training. The internationally comparative view proved to be especially helpful for addressing theoretical issues like “social determinants of health”, “health systems and financing” or “equity and the human right to health“. Global Health means to think local, to ask for the local meaning of globally relevant issues like health inequities, and to reflect on the agency, individual responsibilities and personal scope of action in any particular situation and site. The focus and benefits of global health education are not confined to situations “abroad”. Students also get new insight into the situation “at home” like in Germany, were the globally relevant topics mentioned above are equally relevant and deserve attention. Students obtain a better understanding of the strength and weakness of the health system of their home countries compared to others. They frequently report that the broad and interdisciplinary perspective of the SPC, and the comparatively wide space for discussions and reflection, are considered helpful to develop and to defend their personal standpoint and to think about their professional future, generally in Germany. Interdisciplinarity with a strong commitment to social sciences, history and anthropology, broad space for discussion and reflection, and a “global” perspective that starts “at home” are, in our opinion, essential condition for assisting “students to become critical and reflective beings in an interconnected and complex local–global environment” ([29]: 2f).
5. The integration of international elements into the general medical curriculum has been achieved only to a certain extent as yet. The SPC on Global Health still remains an additional program for particularly interested and dedicated students. However, a much larger number of students than those inscribed in the SPC have been reached at least partially. The majority of individual events (lectures etc.), all elective courses and the seminars pre and post international clerkships always include more than the registered students. Looking into the future, a further step might be to integrate single teaching units with international perspective and students’ reports of experiences abroad into clinical and other courses of the general curriculum, e.g. regarding health and migration, or health systems.
6. Regarding the possibilities to transfer the Giessen model to other universities, the major principle is: *“think global - act local“*. The structure of the SPC is based on the specific conditions at the universities of Giessen and Marburg. The elements and didactic principles for global health education presented in this article and many others can, however, be adapted to the local conditions at any other academic center (cf. [6], [7], [5], [8], [14], [18], [29]). Based on a suitable definition of Global Health, pre-existing and newly developed courses and teaching elements can be combined in a locally suitable way. There is not one model but many, illustrated by the huge variety of courses recently developed in Germany: Various universities are now offering courses on Global Health, all adapted to the particular local structures, priorities and possibilities: Aachen, Berlin, Bonn, Freiburg, Greifswald, Hamburg, Heidelberg, Marburg, Munich, Ulm, and Wurzburg. Innovative and high quality teaching depends, however, on resources. Global Health education that actually complies with the standards and goals expressed e.g. by the HRK [16] is only possible when faculties and universities provide appropriate and sustainable resources – not only for student mobility but also for organizing and offering the students a suitable and conceptually sound didactic frame.

## Conclusions

Prof. Margret Wintermantel, former president of the HRK and currently of the German Academic Exchange Service (*Deutscher Akademischer Austauschdienst*, DAAD), stated that today, universities should prepare students to “live and work in different countries and foreign cultures, and to assume civil responsibilities” ([16]: 5). In the case of medicine, coherent educational programs that systematically connect international activities and exchange to current debates on global health education might be an important contribution to achieve this goal. Internationalizing medical education means more than only increasing the numbers of students going abroad. 

Internationalizing medical education requires a broad array of perspectives and scientific disciplines that transcend the boundaries of clinical and biomedical disciplines, tropical medicine and public health. These remain of upmost importance, yet need to be embedded in a truly interdisciplinary framework with an equally strong and scientifically serious consideration of the social, cultural, political, historical and moral dimensions of health and medical care within the dynamics between local realities and global context. How, if not using the perspectives and means of social sciences and the humanities, can the non-biological aspects of medicine and health be addressed? How, if not by using theories, methods and knowledge of social sciences and the humanities might issues like cultural competence and cross-cultural understanding be taught to avoid misunderstandings, misinterpretation and discrimination (cf. [2], [11], [19], [21])? Applying social theories and a historically informed, critical and reflective perspective on medicine, global and local health is essential for achieving the ambitious goals of internationalizing medical education, and for making best use of its huge didactic potential (cf. [1], [4], [7], [20], [27]).

The SPC Global Health is one project for the internationalization of medical education, adapted to the particular local conditions at Justus Liebig University Giessen. At any other university, different approaches have to be developed to “think global and teach local”.

## Competing interests

The authors declare that they have no competing interests.

## Figures and Tables

**Table 1:**
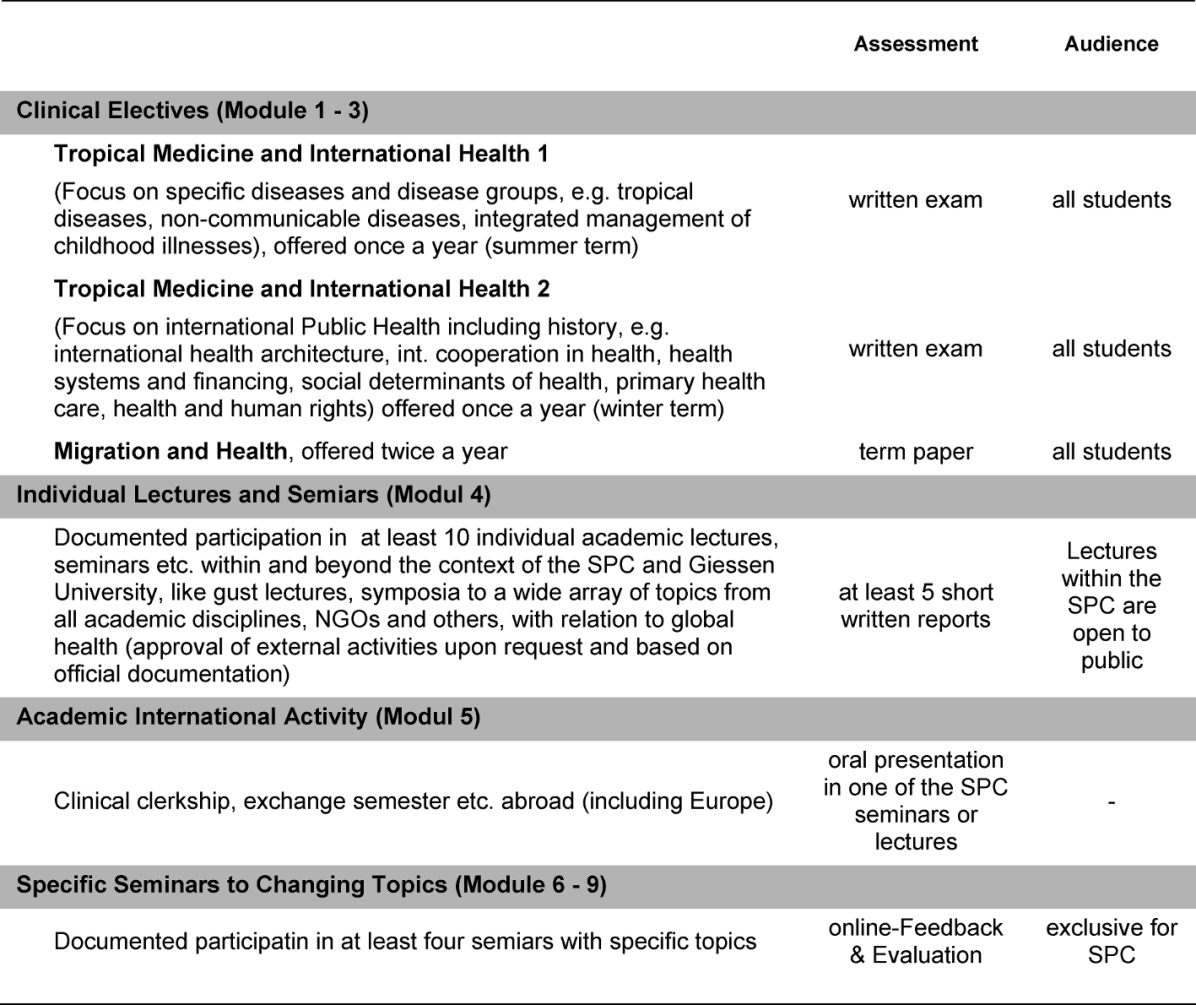
Elements oft the SPC Global Health (Portfolio of 9 modules)

**Table 2:**
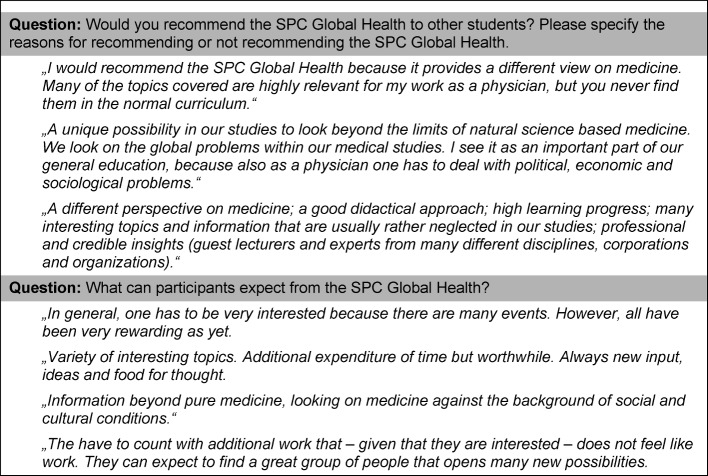
Characteristic quotes from answers to open questions, winter term 2011/12 (translated from German)
